# Enhancement of Oxytocin in the Medial Prefrontal Cortex Reverses Behavioral Deficits Induced by Repeated Ketamine Administration in Mice

**DOI:** 10.3389/fnins.2021.723064

**Published:** 2021-09-10

**Authors:** Weili Zhu, Zengbo Ding, Zhihui Zhang, Xiao Wu, Xiaoya Liu, Ya Zhang, Suxia Li, Liping Zhou, Geng Tian, Jing Qin

**Affiliations:** ^1^National Institute on Drug Dependence, Peking University & Beijing Key Laboratory of Drug Dependence, Peking University, Beijing, China; ^2^Department of Stomatology, Peking University Third Hospital, Beijing, China; ^3^Precision Medicine Research Center, School of Pharmacy, Binzhou Medical University, Yantai, China; ^4^Department of Pharmaceutics, School of Pharmacy, Fudan University & Key Laboratory of Smart Drug Delivery, Ministry of Education, Shanghai, China

**Keywords:** oxytocin, ketamine, medial prefrontal cortex, social avoidance, cognitive impairment, inflammatory mediators, immune markers

## Abstract

Ketamine is a popular recreational substance of abuse that induces persistent behavioral deficits. Although disrupted oxytocinergic systems have been considered to modulate vulnerability to developing drugs of abuse, the involvement of central oxytocin in behavioral abnormalities caused by chronic ketamine has remained largely unknown. Herein, we aimed to investigate the potential role of oxytocin in the medial prefrontal cortex (mPFC) in social avoidance and cognitive impairment resulting from repeated ketamine administration in mice. We found that ketamine injection (5 mg/kg, i.p.) for 10 days followed by a 6-day withdrawal period induced behavioral disturbances in social interaction and cognitive performance, as well as reduced oxytocin levels both at the periphery and in the mPFC. Repeated ketamine exposure also inhibited mPFC neuronal activity as measured by a decrease in c-fos-positive cells. Furthermore, direct microinjection of oxytocin into the mPFC reversed the social avoidance and cognitive impairment following chronic ketamine exposure. In addition, oxytocin administration normalized ketamine-induced inflammatory cytokines including TNF-α, IL-6, and IL-1β levels. Moreover, the activation of immune markers such as neutrophils and monocytes, by ketamine was restored in oxytocin-treated mice. Finally, the reversal effects of oxytocin on behavioral performance were blocked by pre-infusion of the oxytocin receptor antagonist atosiban into the mPFC. These results demonstrate that enhancing oxytocin signaling in the mPFC is a potential pathway to reverse social avoidance and cognitive impairment caused by ketamine, partly through inhibition of inflammatory stimulation.

## Introduction

Ketamine abuse has become a global issue, although it was originally developed as a dissociative anesthetic for medical purposes. For the past two decades, ketamine has been a popular recreational substance of abuse due to its hallucinogenic and addictive properties, leading to major risks and challenges to public health worldwide (Sassano-Higgins et al., 2016). However, the mental and psychological profiles of ketamine, particularly the neurocognition potentials, are quite underestimated relative to those of traditional drugs. Hence, it is important to be able to evaluate the neurobiological processes that are relevant to ketamine exposure-encoded behavioral phenotypes, including social avoidance and cognitive deficits, which are associated with an increased risk of drug relapse. Therefore, we must clarify whether neuropeptide signaling pathways and neuronal activity in specific brain regions are involved in the behavioral and cognitive changes caused by ketamine.

Mounting evidence has indicated that the neuropeptide oxytocin modulates vulnerability to developing drugs of abuse (Bowen and Neumann, 2017), suggesting the possibility of targeting oxytocin signaling for the treatment of drug dependence (McGregor and Bowen, 2012). Ketamine abusers showed significantly decreased blood levels of oxytocin compared with healthy controls, without restoration even after 2 weeks of abstinence (Huang et al., 2018). Previous studies have shown that oxytocin is associated with neurobehavioral processes, including social recognition and cognitive performance (Popik et al., 1992; Feifel et al., 2016). Social avoidance, as seen in decreased contacts and time with partners in the social interaction, and impaired cognitive function are characteristic behavioral changes induced by ketamine (Lipska and Weinberger, 2000; Gama et al., 2012). However, the social interaction is a protective factor to prevent drug relapse with increased social support and positive coping against drug effects in several rodent models (Venniro et al., 2018; Sampedro-Piquero et al., 2019). Moreover, addictive drug abuse and withdrawal also caused impairments in the social interaction behavior through the enhancement of ketamine's rewarding effects and deficits in social recognition (Liao et al., 2018), suggesting that behavioral changes might partially explain the increased risk of relapse in drug abusers. Meanwhile, social avoidance and cognitive deficits are observed in drug dependence and relapse (Albein-Urios et al., 2019). Therefore, disturbed oxytocin may participate in the pathophysiology of ketamine-induced neurobehavioral phenotypes.

It has been shown that ketamine-induced cognitive dysfunction is associated with increased levels of inflammatory cytokines, such as TNF-α (Sedky and Magdy, 2021). In addition, the counts of immune cell neutrophils and monocytes, as well as the neutrophil–lymphocyte ratio (NLR), reliable inflammatory biomarkers for systemic inflammatory response (Gibson et al., 2007; Azab et al., 2011), were found increased in ketamine and psychiatric disorders with a core symptom of impaired social behaviors (Aydin Sunbul et al., 2017; Kayhan et al., 2017; Kido et al., 2019). These findings suggest that novel pharmacotherapies targeting neuroinflammatory processes may result in improvements in behavioral dysfunctions induced by addictive drugs. Based on these previous studies, we aimed to investigate the potential role of oxytocin in the medial prefrontal cortex (mPFC) in the social avoidance and cognitive impairment resulting from repeated ketamine administration in mice and the possible involvement of inflammatory stimulation. We chose the mPFC because of its critical role in both inflammatory response (Costa-Pinto et al., 2005; Tonelli et al., 2009) and social behaviors, as well as its rich expression of oxytocin receptors in rodents (Smeltzer et al., 2006; Li et al., 2016).

## Materials and Methods

### Animals

Male C57Bl/6 (6–8 weeks old) mice were obtained from the Peking University Experimental Animal Center. The mice were group housed under a standard facility with a constant temperature (23 ± 2°C) and humidity (50 ± 5%) and maintained on a 12 h/12 h light/dark cycle (7 a.m. light on; 7 p.m. light off) with *ad libitum* food and water access. All of the procedures were performed with the approval from the Animal Experimentation Ethics Committee of Peking University and in accordance with the National Institutes of Health Guide for the Care and Use of Laboratory Animals (Approval No.: LA2018314 and Approval time: October 26, 2018). All of the behavioral tests, drug administrations, and tissue collections were performed during the active phase of the animals.

### Ketamine Treatment and Withdrawal Procedure

Ketamine (provided by the Drug Intelligence and Forensic Center, Ministry of Public Security, purity >99%) was dissolved in fresh, sterile 0.9% saline before the experiment and then diluted to the required volume. The procedure for chronic ketamine administration and withdrawal was based on a previous study (Jacobskind et al., 2018). Ketamine was administered intraperitoneally (*i.p*.) in a volume of 0.2 ml/10 g bodyweight once daily for 10 days in mice, and the dose of 5 mg/kg was referred to a previous publication (Kumbol et al., 2018) and our pilot study. Control animals received 0.9% saline with the same injection volume as that in the ketamine group. After a 10-day ketamine administration, the mice were exposed to a withdrawal period of 6 days. On day 17, the open field and social interaction tests were performed; on day 18, the NORT was conducted, respectively.

### Locomotor Activity Test

To exclude the possible effect of ketamine on the overall locomotion that may affect the social interaction data, we conducted locomotor activity using an activity-monitoring system in the apparatus (42 × 42 × 42 cm) 1 day after a 6-day withdrawal period of ketamine on day 17. Each mouse was placed in the center of the open field and monitored for 5 min, during which the total distance (cm) traveled was recorded by a video camera to evaluate locomotor activity.

### Social Interaction Test

Social interaction test was performed on the next day after 6-day withdrawal of ketamine following previously established protocols (Zhang et al., 2016). Briefly, during the social interaction test, C57Bl/6 mice were placed in an open field equipment (42 × 42 × 42 cm) with a small empty Plexiglas cage (3 × 6 × 25 cm) placed on the middle of one wall. In this time that the animal spent in the area around this cage in which there was no partner, C57Bl/6 mouse was measured over 2.5 min. After a 1-min interval, a C57Bl/6 mouse was introduced into the Plexiglas cage full of small holes, and the procedure was repeated. The time (seconds) the mice spent in the interaction zone with a C57Bl/6 mouse (with partner) or without a C57Bl/6 mouse (no partner) was recorded. The social interaction ratio = time in the interaction zone with a mouse (with partner)/time in the interaction zone without a mouse (no partner).

### Novel Object Recognition Test

The novel object recognition test (NORT) was used to assess the nature of mice to explore novel or unfamiliar objects and differentiate them from those that they were already familiar with (Ennaceur and Delacour, 1987). A day before the training session, the mice were allowed to explore an open-field arena (42 × 42 × 42 cm) without objects for 5 min 1 day before the training session. The next day, two identical objects were placed in opposite and symmetrical corners of the arena. The mice were then placed in the center of the arena with their backs to the objects for 5 min (training session) and, thereafter, returned to their home cages. After 1 h, one of the familiar objects (a yellow cylinder) was replaced with a different one (a green cube); the mice were returned into the arena again and were allowed to explore the objects for 5 min (testing session), in which the time that the mice spent with both objects was recorded. Data were presented as recognition ratio = time (seconds) spent with the novel object ÷ (time spent with the novel object + time spent with the familiar object) (Wang et al., 2007).

### Enzyme-Linked Immunosorbent Assays for Oxytocin in Blood and the Medial Prefrontal Cortex

To measure the plasma oxytocin levels, blood was collected from the inferior vena cava of each mouse and centrifuged (4,000 rpm, 15 min, 4°C) to obtain plasma. Oxytocin levels were determined using an enzyme-linked immunosorbent assay (ELISA) kit (Product Number: ADI-900-153A, Enzo Life Sciences, Inc., New York, NY, USA) following the instructions of the manufacturer. The plasma sample of each mouse was assayed in duplicate, and the mean of the two values was used for analysis. The intra- and inter-assay precisions were 11 and 16%, respectively. The detection range of the oxytocin assay was 15.6–1,000 pg/ml.

For the assessment of oxytocin levels in the mPFC using ELISA, the mouse brains were quickly extracted, frozen in −60°C N-hexane, and transferred to a −80°C freezer. Using a freezing cryostat (−20°C, Reichert-Jung 2800 Frigocut E), bilateral tissue punches (8 gauge) of the mPFC were taken from 1-mm-thick coronal sections approximately 1.70 mm from the bregma, following stereotaxic coordinates. The tissue punches were diluted with the assay buffer in the ELISA kit and homogenized using a high-throughput tissue homogenizer (Smith and Wang, 2014). The tissue homogenate was centrifuged for 15 min at 4°C and 13,000 × *g*, and the supernatant was collected. The brain tissue extraction oxytocin concentrations were measured using the oxytocin ELISA kit, following the instructions of the manufacturer. The total oxytocin content of each sample was normalized by the tissue weight. The plate was read at an optical density of 405 nm using a microplate reader, and the data were calculated from a four-parameter logistic curve fit using ELISA Clac software.

### c-fos Immunohistochemistry

On the next day after the behavioral test, the mice were anesthetized with sodium pentobarbital (60 mg/kg, *i.p*.) and were transcardially perfused with PBS followed by 4% paraformaldehyde. Immunofluorescence procedure was performed as previously described (Xue et al., 2014). The mouse brains were removed and immediately postfixed for 6 h. Fixed brain tissues were placed in 15–20–30% gradient sucrose solution in phosphate buffer saline (PBS). After the dehydration of the sucrose, the brain tissues were rapidly frozen in −65°C N-hexane. The brains were then sectioned coronally with a microtome into 15-μm-thick sections. All of the sections were incubated for 60 min at 37°C in a blocking solution (3% bovine serum albumin and 0.2% Triton X-100 in PBS, pH 7.4). The sections were incubated for 18–24 h at 4°C with monoclonal primary mouse-antibody c-fos (1:1,000; ab208942, Abcam, USA) in the blocking solution. All sections were washed three times in PBS, stained with goat anti-mouse IgG H&L secondary antibody (1:500; Alexa Fluor® 488, ab150117, Abcam, USA), and incubated for 2–4 h. The cell counts on either side of the specific mPFC region were averaged and taken as the positive immunoreactive cell count for each mouse. The number of fluorescent-labeled cells was measured using a fluorescence microscope (Olympus) with an image-analysis program (MetaMorph, version 4.65). For c-fos analysis, ×10 bilateral images were acquired and were manually quantified as those with intensities higher than the background in the mPFC. Data were expressed as the average number of c-fos+ cells per mm^2^ of each section. All quantifications and analyses were completed by an experimenter who was blinded to the treatment conditions.

### Stereotactic Surgery and Intra-Medial Prefrontal Cortex Microinjection

Mice were anesthetized by sodium pentobarbital (Merck KGaA, Darmstadt, Germany, Batch No. 921019, 60 mg/kg, *i.p*.,) before guide cannulae (OD 0.41 mm × ID 0.25 mm) were implanted into their brains using the following stereotaxic coordinates for mPFC: anterior/posterior, +1.75 mm; medial/lateral, ±0.75 mm; dorsal/ventral, −2.65 mm at a 15° angle (Zhang et al., 2016). After surgery, the general health conditions of all mice were monitored, and they were allowed a 7-day recovery period before ketamine treatment. Mice were intracranially microinjected with either oxytocin (Biorbyt, orb71832, 5 ng/μl) or its vehicle (saline) using 10-μl Hamilton syringes (Hamilton, Reno, NV, USA) that were connected via polyethylene-50 tubing (OD 0.61 × ID 0.28) to injectors (OD 0.21 × ID 0.11, RWD Lifescience, Shenzhen, China) extending 0.8 mm beyond the tip of the cannula. A total volume of 0.2 μl oxytocin was infused into the mPFC over 5 min, and the injection syringe was left in place for an additional 5 min to allow for diffusion. For intra-mPFC infusion, oxytocin or its vehicle (saline) was infused once daily for 6 days during the ketamine withdrawal period at a dose of 1 ng/side/mouse referring to a previous report (Kovacs and Marko, 1993). To explore the antagonism of oxytocin receptors, atosiban (MCE, 2.5 ng/μl) or its vehicle (artificial cerebrospinal fluid, 145 mM NaCl, 2.8 mM KCl, 1.2 mM CaCl2, 1.2 mM MgCl2, 5.4 mM D-glucose, pH = 7.4) was infused into the mPFC 30 min before daily oxytocin treatment, and a total volume of 0.2 μl was infused into the mPFC over 5 min. Data from mice with incorrect placements (8 out of 78 mice) assessed using Nissl staining of the bilateral injection cannula were excluded from the statistical analysis.

### Measurements of Cytokines TNF-α, IL-6, and IL-1β Levels in Plasma and the Medial Prefrontal Cortex

For cytokine measurements, plasma was collected using the same procedure as for oxytocin measurement. The tissues of mPFC were weighed and homogenized in PBS by centrifugation for 5 min at 5,000 × g at 4°C, and the supernatant was collected. The levels of cytokines (TNF-α, IL-6, and IL-1β) in the plasma and mPFC extracts were analyzed using commercially available ELISA kits, according to the instructions of the manufacturer. The ELISA Kit of mouse TNF-α (MM-0132M2), mouse IL-6 (MM-0163M2), and mouse IL-1β (MM-0040M2) were purchased from Jiangsu Meimian Biological Technology Co. Ltd (Jiangsu, China).

### Measurements of Neutrophil, Monocyte, and Lymphocyte Counts

Mice were anesthetized with chloral hydrate (5.0%), and 1-ml syringes were immersed in EDTA-2Na before blood collection. Blood was collected from the inferior vena cava using 1-ml syringes and immediately transferred to the EDTA-2K anticoagulant tubes (IDEXX Vet Collect^TM^). The blood samples were analyzed using a fully automatic hematology analyzer (IDEXX procyte DX^TM^) within 6 h after blood collection (Ahmad et al., 2019). The neutrophil to lymphocyte ratio (NLR) and monocyte to lymphocyte ratio (MLR) were calculated as the ratio of the blood neutrophil or monocyte to lymphocyte counts, respectively.

### Statistical Analysis

Statistical analysis was performed using the Prism software (GraphPad 8, San Diego, CA, USA). Data are presented in the figures as mean ± SEM. Differences between the two groups were assessed using two-tailed *t-*tests. Differences among three or more groups were assessed using one-way ANOVA with *Tukey's* multiple comparisons test. Significant differences are indicated in the figures by ^*^p < 0.05, ^**^p < 0.01, ^***^p < 0.001, and ^****^p < 0.0001.

## Results

### The Behavioral Deficits Induced by Repeated Ketamine Administration Is Associated With Impaired Oxytocin Function

**Figure 1:**
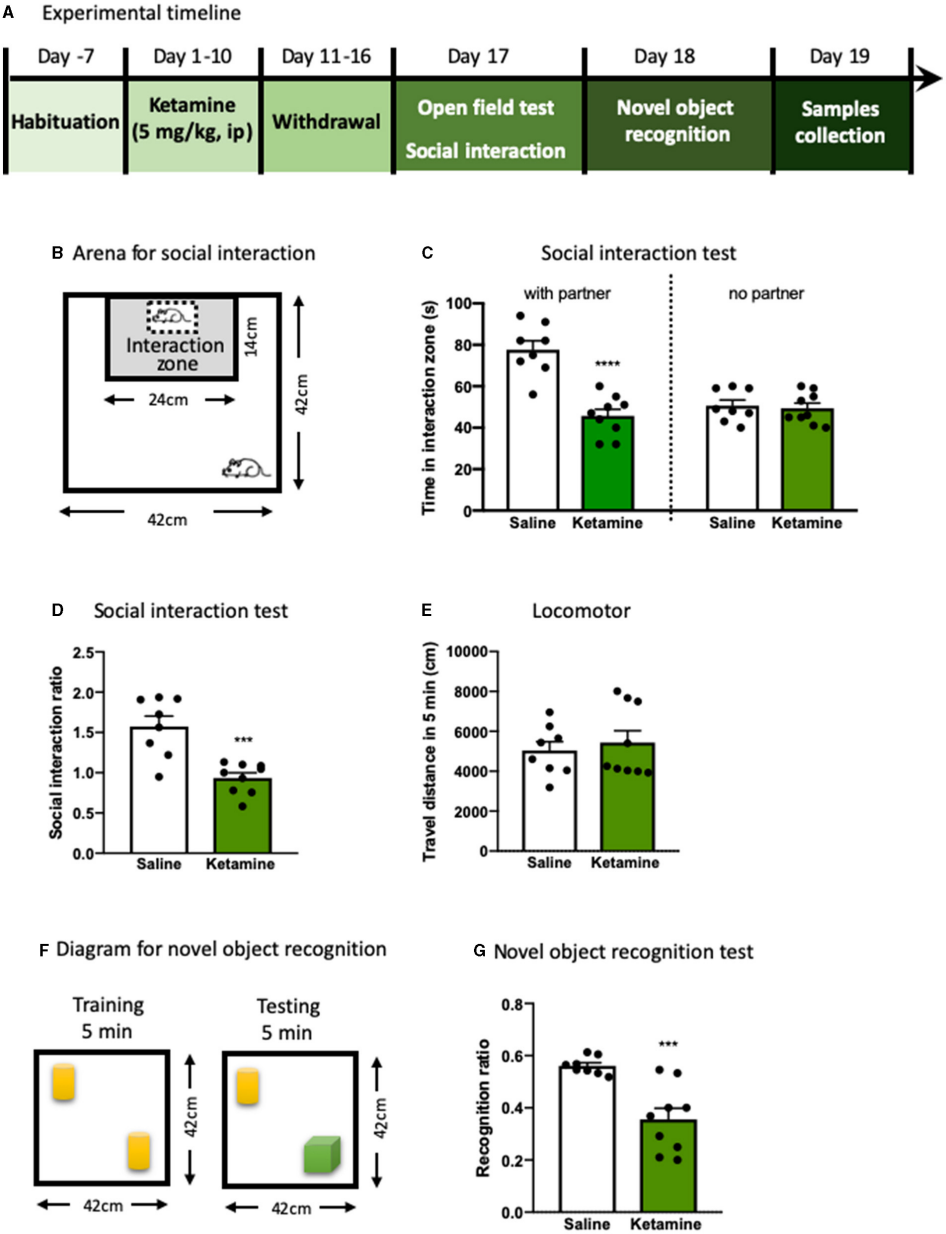
The behavioral deficits induced by repeated ketamine administration. **(A)** Experimental timeline of ketamine treatment and behavioral protocol. **(B)** The graphical scheme of arena for social interaction test. **(C)** The time spent in the social interaction zone. **(D)** The social interaction ratio measured in social interaction test. **(E)** Total distance traveled during the 5- min open field test. **(F)** The diagram for novel object recognition test. **(G)** The recognition ratio measured in the novel object recognition test. Data are presented as mean ± SEM ***p < 0.001, ****p < 0.0001, compared with saline group. *n* = 8–9 per group.

**Figure 2:**
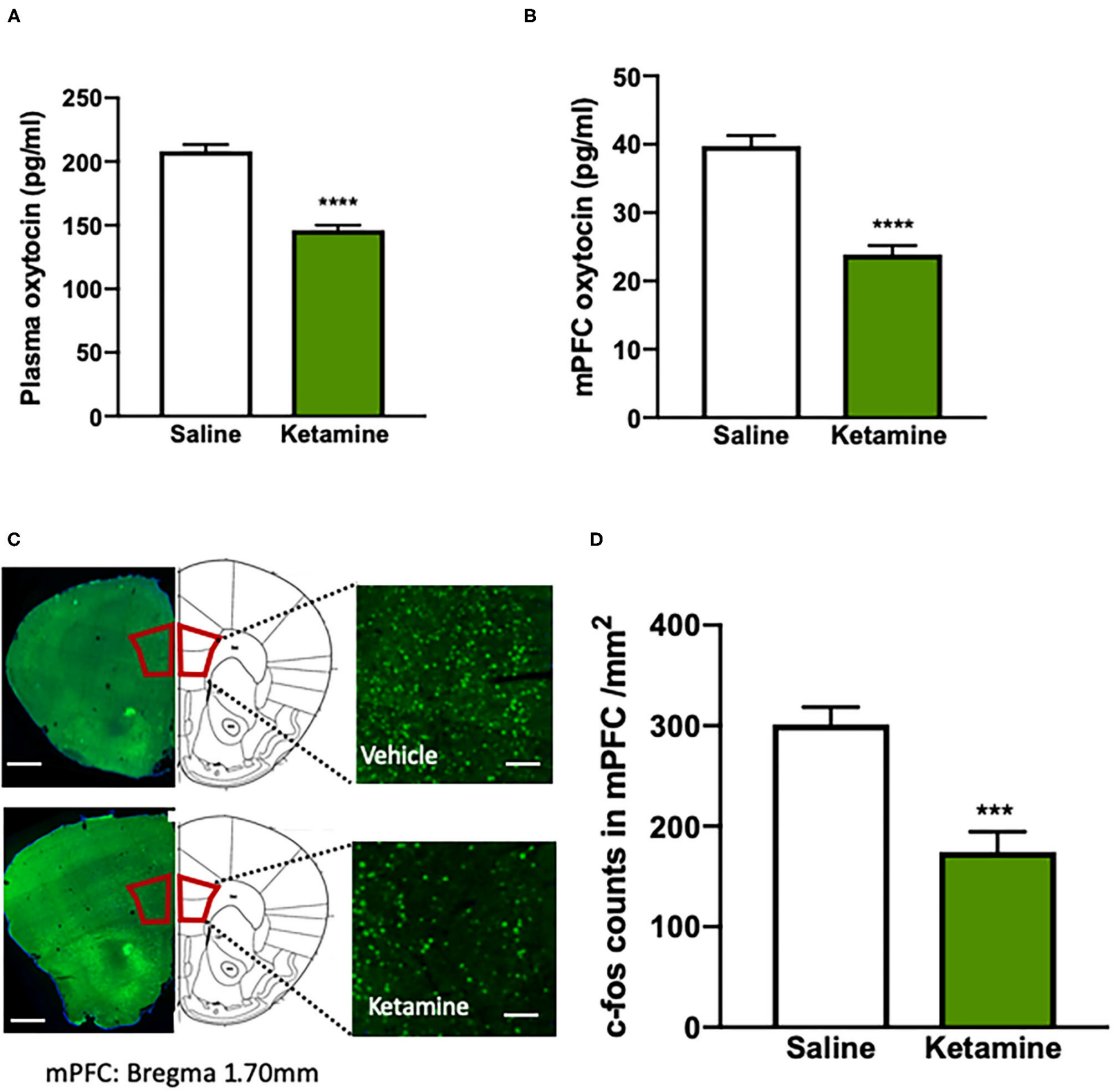
Impaired oxytocin function is associated with the behavioral deficits induced by repeated ketamine administration. **(A)** The levels of plasma oxytocin, and **(B)** the levels of medial prefrontal cortex (mPFC) oxytocin measured by enzyme-linked immunosorbent assay (ELISA). ****p < 0.0001, compared with saline group. *n* = 8–9 per group. **(C)** The representative photographs of the c-fos-positive cell staining with immunohistochemistry and coronal brain sections in the mPFC. Scale bars indicate 0.5 mm (left images) and 100 μm (right images). **(D)** The number of fluorescent-labeled c-fos^+^ cells for each of the quantified regions (four to six images per mouse, standardized to 1 mm^2^). Data are presented as mean ± SEM. ***p < 0.001, ****p < 0.0001 compared with saline group. *n* = 6 per group.

To test the effect of exposure to ketamine on the social behavior and cognitive function, we used social interaction and NORT after a consecutive 10-day ketamine treatment followed by a 6-day withdrawal period (Figure 1A). Mice that received ketamine injection exhibited a significant reduction in social interaction compared with saline-treated mice; this was shown as a reduction in both time in the social interaction zone (*p* < 0.0001, Figures 1B,C) and social interaction ratio (*p* < 0.001, Figure 1D) without alterations in locomotor activity (Figure 1E), suggesting that repeated ketamine administration and withdrawal-induced social avoidance in mice. In the NORT, ketamine-treated mice showed a significantly decreased recognition ratio compared with mice in the saline group (*p* < 0.001, Figures 1F,G). These findings reveal that ketamine-treated mice exhibit behavioral deficits, including social avoidance and cognitive impairment. We further found that exposure to ketamine after withdrawal induced a robust decrease in oxytocin levels in both the plasma (*p* < 0.0001, Figure 2A) and the mPFC (*p* < 0.0001, Figure 2B). Since the normal physiological activity of the mPFC is involved in the development of the social and cognitive processes, we next measured the neuronal activity of the mPFC. Data analyses from the immunohistochemistry assay of c-fos staining in the mPFC showed that c-fos-positive cells in the ketamine-injected group were higher than those in the saline group (*p* < 0.001, Figures 2C,D). These results indicate that the behavioral deficits induced by repeated ketamine administration might be associated with a decrease in oxytocin signaling and neuronal activity in the mPFC.

### Microinjection of Oxytocin Into Medial Prefrontal Cortex Reverses the Behavioral Deficits Induced by Repeated Ketamine Administration

**Figure 3:**
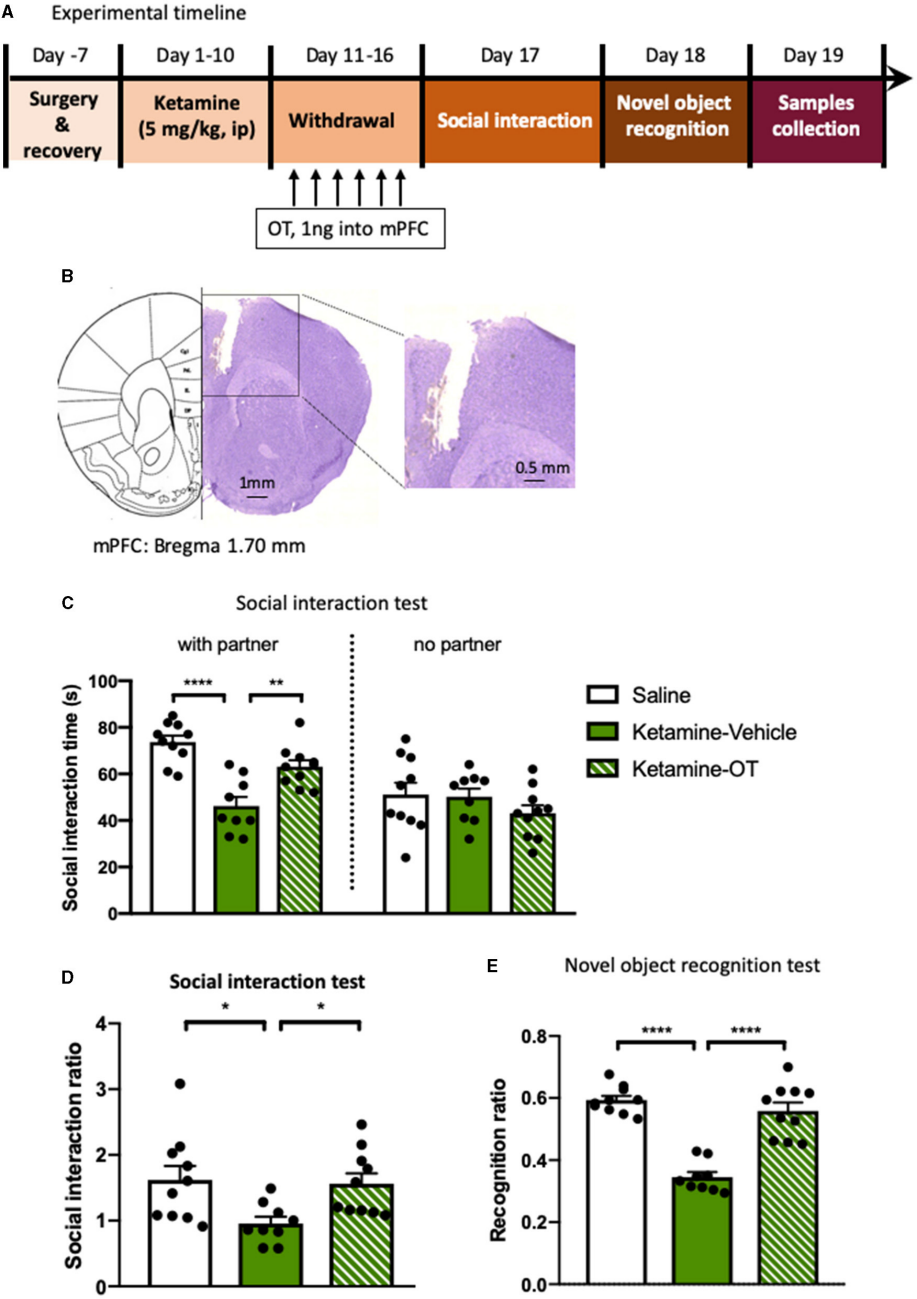
Microinjection of oxytocin into mPFC reverses the behavioral deficits induced by repeated ketamine administration. **(A)** Experimental timeline for ketamine treatment, oxytocin microinjection, and behavioral protocol. **(B)** The indicative photograph of the injection sites of coronal brain sections and representative cannula placements in the mPFC. **(C)** The time spent in the social interaction zone and **(D)** the social interaction ratio measured in social interaction test. **(E)** The recognition ratio measured in the novel object recognition test. Data are presented as mean ± SEM. *p < 0.05, **p < 0.01, ****p < 0.0001 compared with saline or ketamine-vehicle group. *n* = 9–10 per group. OT, oxytocin.

We next examined the effects of oxytocin enhancement in the mPFC on the behavioral deficits induced by repeated ketamine administration. Stereotactic surgery was performed for intra-mPFC microinjection in both ketamine- and saline-treated mice. The mice were then subjected to a 10-day ketamine injection and 6-day withdrawal. Oxytocin (1 ng per side) or its vehicle was microinfused into the mPFC during withdrawal period once daily for 6 days before the social interaction and NORT (Figure 3A). The results showed that administering ketamine induced social avoidance as measured by the social interaction time [*F*_(2, 26)_ = 8.67, *p* < 0.0001, Figures 3B,C] and reduced interaction ratio [*F*_(2, 26)_ = 3.93, *p* < 0.05, Figure 3D], and impaired cognition compared with the saline group [*F*_(2, 26)_ = 12.24, *p* < 0.0001, Figure 3E]. However, mPFC infusions of oxytocin increased the social interaction time [*F*_(2, 26)_ = 5.33, *p* < 0.01, Figure 3C] and social interaction ratio [*F*_(2, 26)_ = 3.59, *p* < 0.05, Figure 3D]. A similar effect was found after the infusion of oxytocin in the mPFC in the NORT, in which the recognition ratio exhibited in the ketamine treatment group was significantly increased by oxytocin infusion [*F*_(2, 26)_ = 10.51, *p* < 0.0001, Figure 3E]. Our data are consistent with previous reports that central administration of oxytocin enhanced certain social behaviors and cognitive capacities (Meyer-Lindenberg et al., 2011). These results indicate that oxytocin in the mPFC mediates the development and reversal of social avoidance and cognitive impairment induced by ketamine.

### Medial Prefrontal Cortex Oxytocin Normalizes Ketamine-Induced Increase in TNF-α, IL-6, and IL-1β Levels

**Figure 4:**
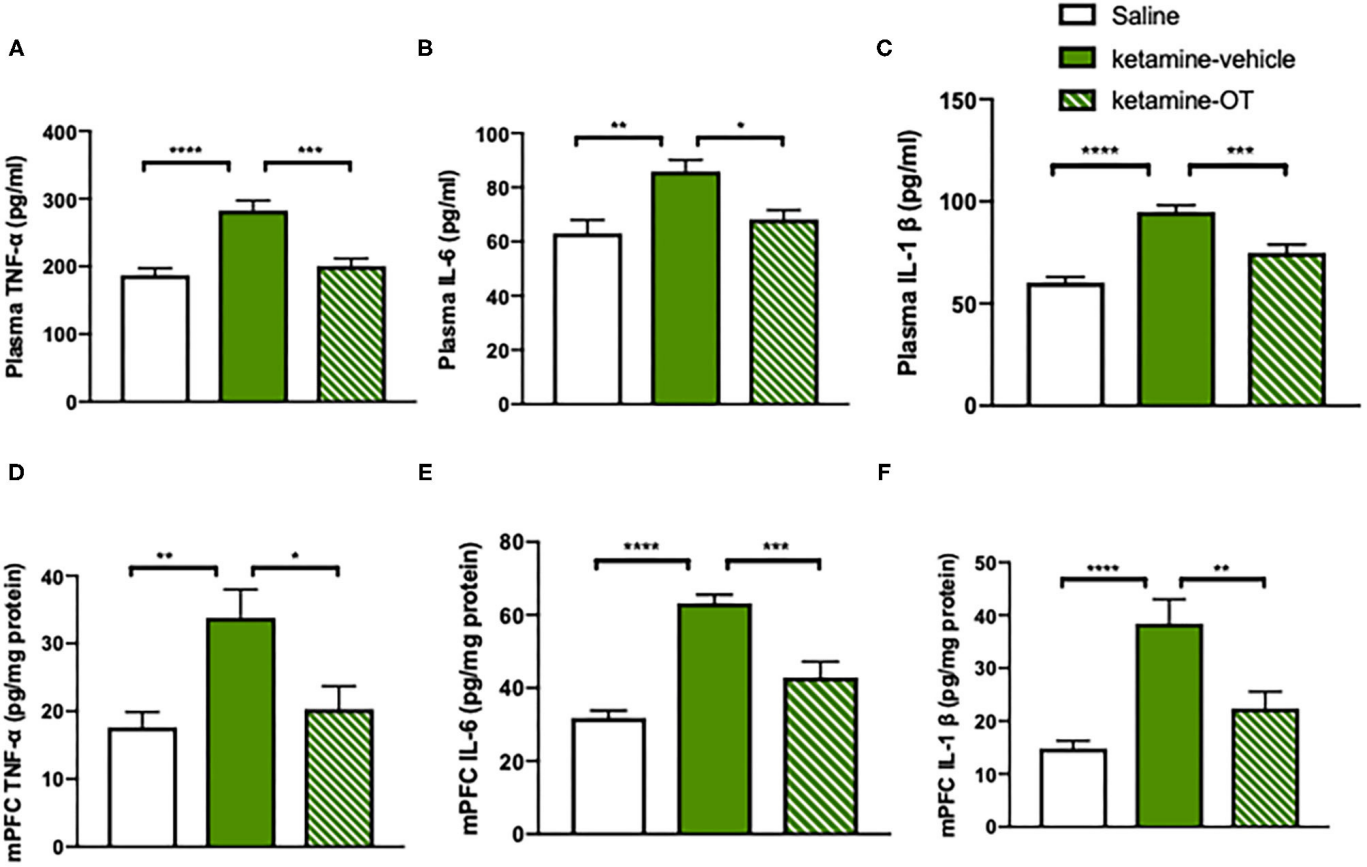
mPFC oxytocin normalizes ketamine-induced cytokines increase in TNF-α, IL-6, and IL-1β levels. **(A)** The levels of TNF-α, **(B)** IL-6, and **(C)** IL-1β in the plasma. **(D)** The levels of TNF-α, **(E)** IL-6, and **(F)** IL-1β in the mPFC measured by ELISA Kit. Data are presented as mean ± SEM. *p < 0.05, **p < 0.01, ***p < 0.001, ****p < 0.0001, compared with saline or ketamine-vehicle group. *n* = 9–10 per group. OT, oxytocin.

We next investigated the potential pathway underlying the beneficial effects of oxytocin on the reversal of social behaviors. Previous studies have shown that the inflammation is involved in the psychosocial behaviors during the drug withdrawal period, with increased levels of TNF-α, IL-6, and IL-1β (Hu et al., 2016). Additionally, the anti-inflammatory effects of oxytocin are linked to its social interactions by decreasing the inflammatory cytokines TNF-α, IL-6, and IL-1β in both rodents and humans (McQuaid et al., 2014). We attempted to provide direct evidence that intra-mPFC oxytocin regulates the inflammatory process that occurs in ketamine-induced behavioral deficits. To achieve this goal, the mice were treated with ketamine for 10 days and exposed to a 6-day withdrawal period, during which the mice were microinjected with oxytocin or its vehicle in the mPFC. On day 17, both blood samples and mPFC brain tissues from mice were collected for ELISA measurements of TNF-α, IL-6, and IL-1β levels. We found that chronic ketamine treatment significantly increased TNF-α [*F*_(2, 26)_ = 7.64, *p* < 0.0001, Figure 4A], IL-6 [*F*_(2, 26)_ = 5.33, *p* < 0.01, Figure 4B], and IL-1β [*F*_(2, 26)_ = 10.12, *p* < 0.0001, Figure 4C] at the periphery, and intra-mPFC oxytocin blocked these activated cytokines of TNF-α [*F*_(2, 26)_ = 6.57, *p* < 0.001, Figure 4A], IL-6 [*F*_(2, 26)_ = 4.13, *p* < 0.05, Figure 4B], and IL-1β [*F*_(2, 26)_ = 5.84, *p* < 0.001, Figure 4C]. Consistently, the levels of TNF-α [*F*_(2, 26)_ = 4.84, *p* < 0.01, Figure 4D], IL-6 [*F*_(2, 26)_ = 9.97, *p* < 0.0001, Figure 4E], and IL-1β [*F*_(2, 26)_ = 7.24, *p* < 0.0001, Figure 4F] in the mPFC were also increased by chronic ketamine, whereas intra-mPFC oxytocin reduced TNF-α [*F*_(2, 26)_ = 4.03, *p* < 0.05, Figure 4D], IL-6 [*F*_(2, 26)_ = 6.45, *p* < 0.001, Figure 4E], and IL-1β levels [*F*_(2, 26)_ = 4.92, *p* < 0.01, Figure 4F]. These results indicated that both circulating and mPFC pro-inflammatory cytokine levels were elevated in ketamine-treated mice. Infusion of oxytocin in the mPFC during the withdrawal period significantly decreased TNF-α [*F*_(2, 26)_ = 4.03, *p* < 0.05, Figure 4D], IL-6 [*F*_(2, 26)_ = 6.45, *p* < 0.001, Figure 4E], and IL-1β [*F*_(2, 26)_ = 4.92, *p* < 0.01, Figure 4F] levels not only in the plasma but also in the mPFC (Figures 4D–F), suggesting an attenuated effect of oxytocin on increased inflammatory factors in mice with ketamine withdrawal.

### Medial Prefrontal Cortex Oxytocin Regulates the Blood Immune Markers in Ketamine-Treated Mice

**Figure 5:**
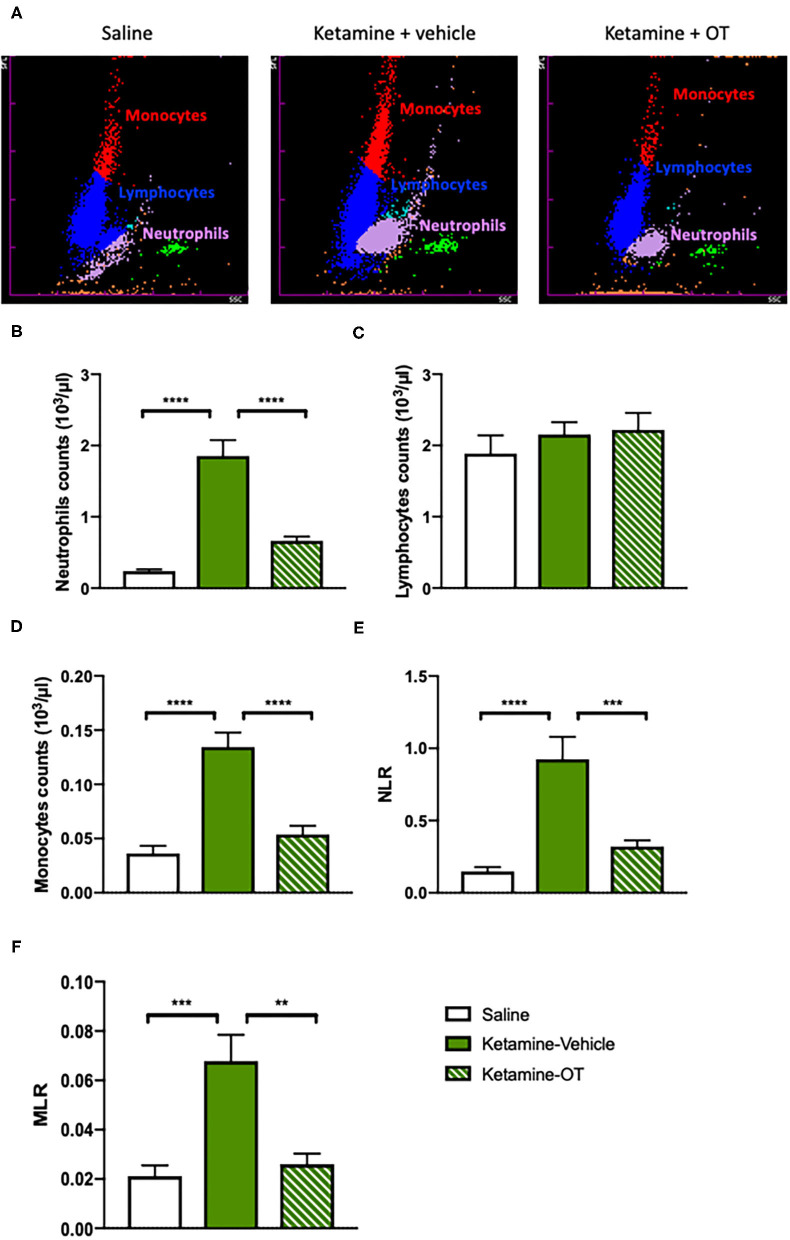
mPFC oxytocin regulates the blood immune markers in ketamine-treated mice. **(A)** The representative images from hematology analyzer showing neutrophils, lymphocytes, and monocytes. **(B)** The count of blood neutrophils, **(C)** lymphocytes, **(D)** monocytes, **(E)** NLR, and **(F)** MLR. Data are presented as mean ± SEM. **p < 0.01, ***p < 0.001, ****p < 0.0001, compared with saline or ketamine-vehicle group. *n* = 8–9 per group. NLR, the neutrophil to lymphocyte ratio; MLR, the monocyte to lymphocyte ratio; OT, oxytocin.

Since it has been evidenced that there is a significant relationship between immune markers and cognitive symptoms or drug abuse (Guzel et al., 2018; Fourrier et al., 2020), we determined the parameters related to the immune system such as neutrophils, lymphocytes, monocytes, NLR (the calculated ratio of the blood neutrophil to lymphocyte count), and MLR (calculated by dividing the monocyte count by the lymphocyte count) (Figure 5A). We found that the neutrophils [*F*_(2, 22)_ = 11.40, *p* < 0.0001, Figure 5B] and monocyte counts [*F*_(2, 22)_ = 9.68, *p* < 0.0001, Figure 5D] increased with chronic ketamine administration, whereas the upregulation of neutrophils [*F*_(2, 22)_ = 8.40, *p* < 0.0001, Figure 5B] and monocytes [*F*_(2, 22)_ = 7.95, *p* < 0.0001, Figure 5D] significantly reduced by intra-mPFC oxytocin treatment. Neither ketamine nor oxytocin showed alterations in the lymphocyte count (*p* > 0.05, Figure 5C). In addition, higher values of NLR [*F*_(2, 22)_ = 7.74, *p* < 0.0001] and MLR [*F*_(2, 22)_ = 6.30, *p* < 0.0001] were observed in the ketamine group than in the saline group, but oxytocin infusion decreased the NLR [*F*_(2, 22)_ = 6.02, *p* < 0.001, Figure 5E] and MLR [*F*_(2, 22)_ = 5.64, *p* < 0.01, Figure 5F]. Our results raised the possibility that there may be a potential relationship between the protective effects of oxytocin on behavioral deficits and the restoration of blood immune biomarkers in mice chronically treated with ketamine.

### Oxytocin Receptor Antagonist Blocks the Reversal Effects of Oxytocin on Behavioral Impairments in Ketamine-Treated Mice

**Figure 6:**
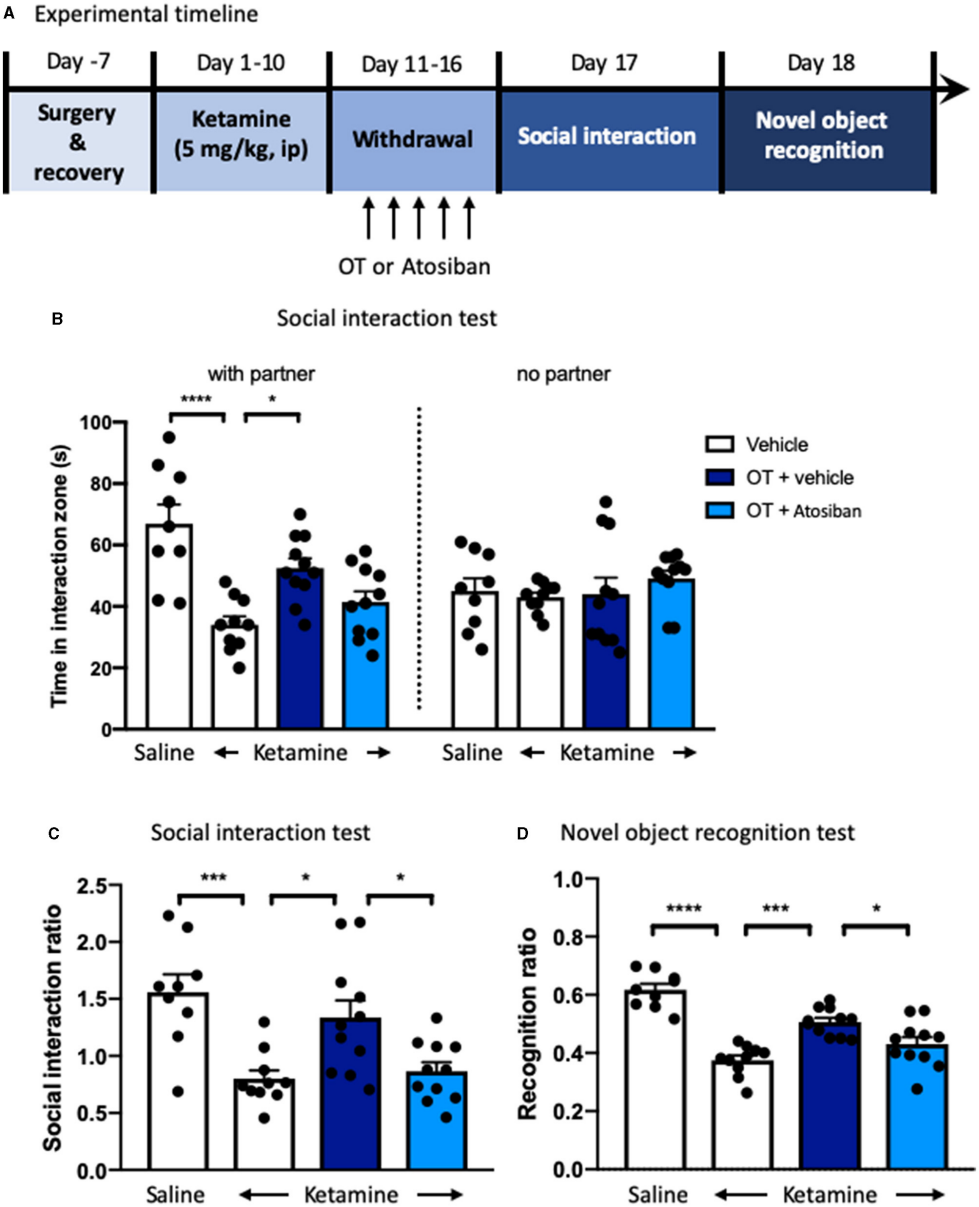
Oxytocin receptor antagonist blocks the reversal effects of oxytocin on behavioral impairments in ketamine-treated mice. **(A)** Experimental timeline for ketamine treatment, oxytocin, or oxytocin receptor antagonist atosiban microinjection, and behavioral protocol. **(B)** The time spent in the social interaction zone, and **(C)** the social interaction ratio measured in the social interaction test. **(D)** The recognition ratio measured in the novel object recognition test. Data are presented as mean ± SEM *p < 0.05, ***p <0.001, ****p < 0.0001, compared with saline, ketamine-vehicle, or ketamine-OT group. *n* = 9–11 per group. OT, oxytocin.

We next examined the role of oxytocin in the mPFC in the behavioral impairment of ketamine withdrawal by the infusion of an oxytocin antagonist atosiban into the mPFC after a 10-day ketamine injection and 6-day abstinence (Figure 6A). Pretreatment with atosiban 30 min before each oxytocin infusion in the mPFC showed a trend of attenuation of the social interaction behavior, which was improved by oxytocin in ketamine-treated mice, although there was no significant difference between groups (Figure 6B). Statistical analyses revealed significant effects of oxytocin [*F*_(3, 37)_ = 4.52, *p* < 0.05, Figure 6C] and atosiban [*F*_(3, 37)_ = 4.07, *p* < 0.05, Figure 6C] on the social interaction ratio. Oxytocin significantly increased the social interaction impaired by chronic ketamine administration, whereas atosiban microinfusion in the mPFC in oxytocin-treated mice reversed the increase in the social interaction ratio. Finally, in the NORT, the recognition ratio reduced by chronic ketamine administration was increased by intra-mPFC oxytocin [*F*_(3, 37)_ = 6.88, *p* < 0.0001, Figure 6D]; however, this increased recognition ratio was hindered by pre-infusion of atosiban into the mPFC [*F*_(3, 37)_ = 4.06, *p* < 0.05, Figure 6D]. Altogether, these results indicate that the enhanced function of oxytocin in the mPFC was associated with improvement of social interaction and cognitive performance in ketamine-treated mice, and antagonism of oxytocin receptors by atosiban in the mPFC blocked the beneficial effects of oxytocin.

## Discussion

**Figure 7:**
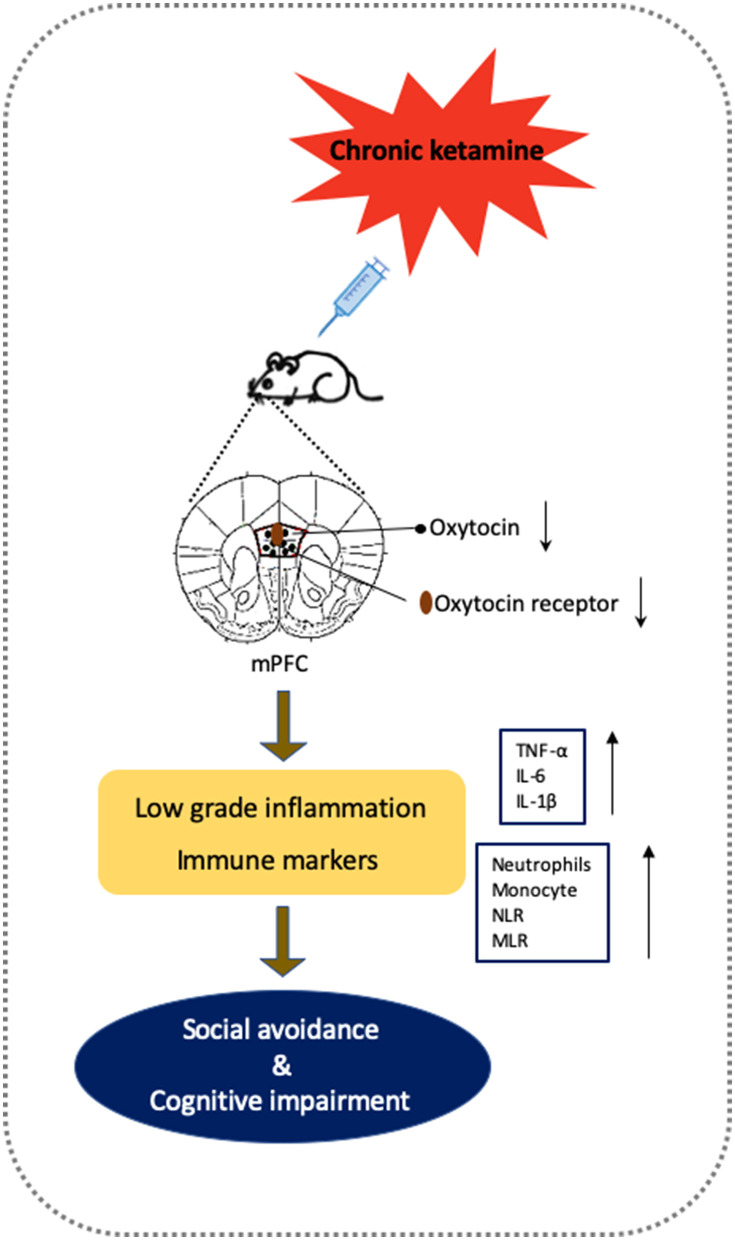
Putative framework of oxytocin on the social avoidance and cognitive dysfunctions induced by chronic ketamine exposure and withdrawal according to experimental findings. Chronic ketamine exposure decreases oxytocin activity, which impairs mPFC function. Chronic mPFC dysfunction in vulnerable animals increases low-grade inflammation (shown by elevated inflammatory cytokines TNF-α, IL-6 <, and IL-1β) and altered immune markers (increased neutrophils, monocyte, NLR, and MLR), producing social avoidance and cognitive impairment. Supplementary oxytocin protects mPFC and inflammatory processes from damage by ketamine to reverse behavioral deficits.

The results of the present study showed that repeated ketamine administration (5 mg/kg, *i.p*.) for 10 days and withdrawal for 6 days induced behavioral deficits in both social interaction and cognitive performance as well as reduced oxytocin levels in the mPFC. Furthermore, direct microinjection of oxytocin into the mPFC reversed the social avoidance and cognitive impairment, whereas pre-infusion of the oxytocin receptor antagonist atosiban blocked the reversal effects of oxytocin. Repeated ketamine exposure also inhibited mPFC neuronal activity as measured by a decrease in c-fos-positive cells. In addition, oxytocin administration normalized ketamine-induced inflammatory cytokines including TNF-α, IL-6, and IL-1β levels both at the periphery and in the mPFC. Finally, the activation of immune markers such as neutrophils and monocytes by ketamine was restored in oxytocin-treated mice. Taken together, these results demonstrate that enhancing oxytocin signaling in the mPFC is a potential pathway to reverse social avoidance and cognitive impairment via normalization of inflammatory mediators as well as immune markers and may represent a promising therapeutic strategy for treating ketamine-induced behavioral disturbances (Figure 7).

Oxytocin, synthesized in the magnocellular neurons situated in the supraoptic and paraventricular nuclei of the hypothalamus and processed to the pituitary, has been shown to increase social interaction and improve cognitive performance in mental disorders characterized by social impairments (MacDonald et al., 2013; Teng et al., 2013; Havranek et al., 2015). Given that previous studies prescribed a negative relationship between oxytocin levels and ketamine treatment (Huang et al., 2018), there is a potential for introducing oxytocin as a therapeutic candidate to restore impaired functions in social and cognitive behaviors. As expected, we found from the present study that the plasma oxytocin was significantly decreased by repeated ketamine administration and withdrawal, suggesting that there might be a direct association between the oxytocin system and ketamine-induced social disorders. It is also noteworthy that reduced central oxytocin levels were observed in the mPFC, which is thought to play an essential role in cognition and social behavior (Ferguson and Gao, 2018; Jung et al., 2021). Dysfunction of the mPFC may be responsible for impairments in social interaction and cognitive performance in several studies (Ferguson and Gao, 2018). Importantly, whether the social interaction deficits caused by ketamine could be restored by oxytocin is determined by the pattern of administration. For example, systematic oxytocin did not ameliorate social deficits, contrary to studies in which this deficit was restored by intracerebroventricular oxytocin administration (Havranek et al., 2015). In the present study, we aimed to investigate whether a specific infusion of oxytocin in the mPFC has a positive effect on the social and cognitive functions in mice with ketamine withdrawal. Our results showed that ketamine treatment was associated with a significant decrease in social interactions and cognitive deficits, which is consistent with previous studies (Silvestre et al., 1997). In addition to reduced oxytocin levels in the mPFC, we further found that the neuronal activity in the mPFC was also inhibited by ketamine relative to mice in the control group, suggesting that the improvement of mPFC activity as well as oxytocin function in the mPFC may be necessary for reversal of these social deficits induced by ketamine. We found here that oxytocin microinfused in the mPFC during the withdrawal period reversed the social avoidance and cognitive impairment induced by ketamine. Although the mPFC activity after oxytocin administration has not been investigated in this study, it is noteworthy that the normal function of the mPFC contributes to the neuronal pathways underlying social and cognitive function; hence, we found a potential association between mPFC oxytocin and behavioral improvements. However, it is still necessary to further determine the molecular regulators in this link.

Stressful situation performance is considered to be responsible for the ketamine abstinence-induced impairment in social interaction and cognitive performance due to the increased stress hormones that can increase the risk of the development of substance abuse and relapse (Huang et al., 2020). The regulatory role of oxytocin in behavioral responses to stress has also been investigated. For example, both peripheral blood and central levels of oxytocin were elevated by various stressful procedures, whereas administration of oxytocin restored these changes by reducing stress adrenocorticotropic hormone and corticosteroid concentrations (Wotjak et al., 1998; Windle et al., 2004). Regarding the link between oxytocin function and stress systems, numerous studies support the hypothesis that oxytocin is generally associated with an active response to stress by inhibiting the hyperactivity of the hypothalamic–pituitary–adrenal (HPA) axis (Kormos and Gaszner, 2013), leading to the mitigation of the negative effects of stress on social and cognitive functions. In addition, stress-induced reinstatement of addictive methamphetamine increased inflammatory cytokines in the prefrontal cortex, leading to changes in neurotransmission, thus, triggering reinstatement of methamphetamine (Karimi-Haghighi et al., 2020). Moreover, the increased pro-inflammatory cytokines (TNF-α, IL-6, and IL-1β) were found in addictive psychostimulants including ketamine, methamphetamine, and alcohol (Airapetov et al., 2019; Li et al., 2020; Sedky and Magdy, 2021), suggesting that changes in peripheral markers of inflammation are generally associated with drug abuse. These data raised the possibility that oxytocin participates in social and cognitive functions through interactions with inflammatory processes, and reduction in inflammatory cytokines may play an important role in the reversal effect of oxytocin on ketamine-induced behavioral dysfunctions. Of note, pro-inflammatory cytokines including IL-6 and IL-1β altered the oxytocin receptor expression in tissues (Baribeau and Anagnostou, 2015), suggesting that reducing inflammatory activity in early abstinence could change social and cognitive deficiencies. Therefore, attenuation of the pro-inflammatory effects of addictive drug administration may have implications on the treatment of drug abuse. In the present study, we found that both circulating and mPFC pro-inflammatory cytokine levels were elevated in ketamine-treated mice, whereas infusion of oxytocin in the mPFC during the withdrawal stage significantly decreased TNF-α, IL-6, and IL-1β levels. These results suggest a protective effect of oxytocin on increased inflammatory factors in mice with ketamine withdrawal.

Together with increased levels of pro-inflammatory cytokines (i.e., TNF-α, IL-1β, and IL-6), ketamine caused redox dysregulations, such as elevated production of reactive oxygen species (ROS) in the prefrontal cortex of adult mice (Bove et al., 2020). It has also been shown that redox dysregulation is significantly implicated in the development of cognitive and social dysfunctions in rodents (Genius et al., 2013). Moreover, oxidative alterations could prevent cognitive and social behavioral deficits induced by ketamine administration in mice (Phensy et al., 2017; Ben-Azu et al., 2018). Hence, ketamine-induced neuroinflammation and consequent ROS release may play a substantial role in the pathophysiology of the behavioral deficits. Further investigations targeting the inhibition of inflammatory pathways and enhancement of antioxidant defense could be helpful in preventing the neurobehavioral abnormalities caused by ketamine.

Regarding the association of immune markers and social interaction, a recent study showed that repeated social defeat stress increased neutrophils and monocytes in the blood, and this increase may contribute to the development of social avoidance (Ishikawa et al., 2021). Additionally, NLR and MLR were significantly associated with cognitive performance in patients with major depressive disorder (Fourrier et al., 2020). However, the role of immune markers particularly neutrophils, lymphocytes, monocytes, NLR, and MLR in social interaction, and cognitive performance improved by oxytocin remains unclear. We found that the neutrophils and monocyte counts as well as NLR and MLR were increased by chronic ketamine injection, and intra-mPFC oxytocin treatment significantly reduced these immune markers. Our results indicate that there is a potential relationship between the protective effects of oxytocin on social and cognitive deficits and the restoration of blood immune biomarkers in mice chronically treated with ketamine. Although several studies have summarized the potential treatment of oxytocin for social deficits and cognitive dysfunction (Meyer-Lindenberg et al., 2011; Baribeau and Anagnostou, 2014), the specific effects of oxytocin on inflammatory processes and the immune system have not been fully identified. Furthermore, investigations are needed to determine whether anti-inflammatory agents can increase oxytocin levels reduced by chronic ketamine treatment to provide a direct evidence supporting the link between inflammation and the social behavior network regulated by oxytocin.

Oxytocin distribution is consistent with the expression pattern of its receptors, which are G protein-coupled receptors that bind to oxytocin, expressed in several regions of the rodent brain, including the mPFC (Shapiro and Insel, 1992; Donovan et al., 2018). The mPFC is known to play a crucial role in both social interaction and cognitive function in a wide range of domains such as social support, positive communication, executive control, learning, and memory. Accordingly, the specific receptor-binding activity is determined by different oxytocin concentrations; the local oxytocin concentrations in the brain are keys for the oxytocin receptor activation and subsequent stimulation of intracellular signaling pathways (Abramova et al., 2020). In the present study, we found that specific antagonism of oxytocin receptors in the mPFC blocked the reversal effects of oxytocin on the social interaction and cognitive performance. Our results further demonstrate that mPFC inactivation of oxytocin signaling pathway by an oxytocin receptor antagonist is critically involved in the behavioral deficits induced by ketamine. The positive effects of oxytocin on behavioral changes are associated with its regulation of neural correlates of the prefrontal cortex, nucleus accumbens, and ventral tegmental area and modulation of the sensitivity to social stimuli with a shared input from oxytocin receptors (Baribeau and Anagnostou, 2015). Moreover, oxytocin was found to enhance the activity in areas including the frontal cortex, nucleus accumbens, and amygdala during social tasks (Gordon et al., 2013). However, it is important to note that the synthetic oxytocin receptor antagonists used in animal studies may not equally affect human physiology; this could be interpreted by the significant genetic variation in the receptor structure across different species (Baribeau and Anagnostou, 2015).

## Conclusion

In conclusion, our results indicate that enhancement of oxytocin in the mPFC has beneficial effects on social avoidance and cognitive deficits induced by chronic ketamine treatment, and withdrawal may be mediated through the regulation of inflammatory cytokines and immune markers. These findings provide a potential strategy for targeting mPFC oxytocin signaling for the prevention of drug withdrawal associated with social and cognitive dysfunctions.

## Data Availability Statement

The original contributions presented in the study are included in the article/supplementary material, further inquiries can be directed to the corresponding author/s.

## Ethics Statement

The animal study was reviewed and approved by the Animal Experimentation Ethics Committee of Peking University.

## Author Contributions

WZ, ZD, JQ, and ZZ conceived and designed the study and wrote the first draft of the study protocol. WZ and GT obtained funding for the study and critically revised the manuscript. ZD and XW contributed to the acquisition of animal data. XL, YZ, LZ, and XW contributed to the acquisition of molecular biology data. ZZ, GT, and SL assisted in the data analysis and interpretation of the data. All authors critically reviewed the content and approved the final version for publication.

## Funding

The project was funded by the National Key Research and Development Program of China (2017YFC0803605 and 2017YFC0803607), the Natural Science Foundation of China (grant nos. 81371489 and 81800983), the Key Basic Research Project of Shandong Provincial Natural Science Foundation (ZR2019ZD27), Peking University Medicine Seed Fund for Interdisciplinary Research (BMU2020MX022 and 71006Y2337), Key Clinical Projects of Peking University Third Hospital (No. BYSYZD2019035), and State Key Laboratory of Environmental Chemistry and Ecotoxicology, Research Center for Eco-Environmental Sciences, Chinese Academy of Sciences (KF2020-18).

## Conflict of Interest

The authors declare that the research was conducted in the absence of any commercial or financial relationships that could be construed as a potential conflict of interest.

## Publisher's Note

All claims expressed in this article are solely those of the authors and do not necessarily represent those of their affiliated organizations, or those of the publisher, the editors and the reviewers. Any product that may be evaluated in this article, or claim that may be made by its manufacturer, is not guaranteed or endorsed by the publisher.
